# Crystal structure of ammonium bis­(pyridine-2,6-di­carboxyl­ato-κ^3^ *O*,*N*,*O*′)chromate(III) from synchrotron data

**DOI:** 10.1107/S2056989015001152

**Published:** 2015-01-24

**Authors:** Dohyun Moon, Jong-Ha Choi

**Affiliations:** a Pohang Accelerator Laboratory, POSTECH, Pohang 790-784, Republic of Korea; b Department of Chemistry, Andong National University, Andong 760-749, Republic of Korea

**Keywords:** Crystal structure, synchrotron radiation, pyridine-2,6-di­carboxyl­ate, ammonium cation, chromate(III) complex, meridional configuration, hydrogen bonding

## Abstract

The structure of the title compound, (NH_4_)[Cr(pydc)_2_] (pydc is pyridine-2,6-di­carboxyl­ate, C_7_H_3_NO_4_), has been determined from synchrotron data. The Cr^III^ ion and the N atom of the ammonium cation are located on a crystallographic fourfold rotoinversion axis (-4). The Cr^III^ cation is coordinated by four O atoms and the two N atoms of two meridional pydc ligands, displaying a distorted octa­hedral geometry. The Cr—N and Cr—O bond lengths are 1.9727 (15) and 1.9889 (9) Å, respectively. The crystal structure is stabilized by inter­molecular hydrogen bonds involving the N–H groups of the ammonium cation and pyridine C–H groups as donors and the non-coordinating carbonyl O atoms as acceptors.

## Abstract 2

In the title complex, the Cr^III^ ion is coordinated by two nearly perpendicular pyridine-2,6-di­carboxyl­ate (pydc) dianions acting as tridentate ligands through the pyridine N atoms and two of the O atoms of each carboxyl­ate group, in a distorted octa­hedral geometry. The ammonium cation is linked to non-coordinating carbonyl O atoms from neighboring pydc groups through N—H⋯O hydrogen-bonding inter­actions.

## Chemical context   

Pyridine-2,6-di­carb­oxy­lic acid (also known as dipicolinic acid and abbreviated here as H_2_pydc) can coordinate a metal center as a neutral mol­ecule (H_2_pydc), the univalent anion (Hpydc^−^), or the divalent anion (pydc^2−^). In particular, the pyridine-2,6-di­carboxyl­ate ligand frequently acts as a merid­ional tridentate ligand and sometimes also as a bidentate or bridging ligand (Park *et al.*, 2007 ▸). The first [Cr(pydc)_2_]^−^ complex was prepared as the Na^+^ salt according to the literature (Hoggard & Schmidtke, 1973 ▸) and its crystal structure determined using synchrotron data. Structural analysis showed the compound to be a dihydrate (Dai *et al.*, 2006 ▸; González-Baró *et al.*, 2008 ▸) rather than the 1.5 or 2.5 hydrates that had been suggested previously (Hoggard & Schmidtke, 1973 ▸; Fürst *et al.*, 1979 ▸). The crystal structures of K[Cr(pydc)_2_] (Hakimi *et al.*, 2012 ▸) and Rb[Cr(pydc)_2_] (Fürst *et al.*, 1979 ▸) have also been reported previously but the structure of the ammonium salt is not known. 
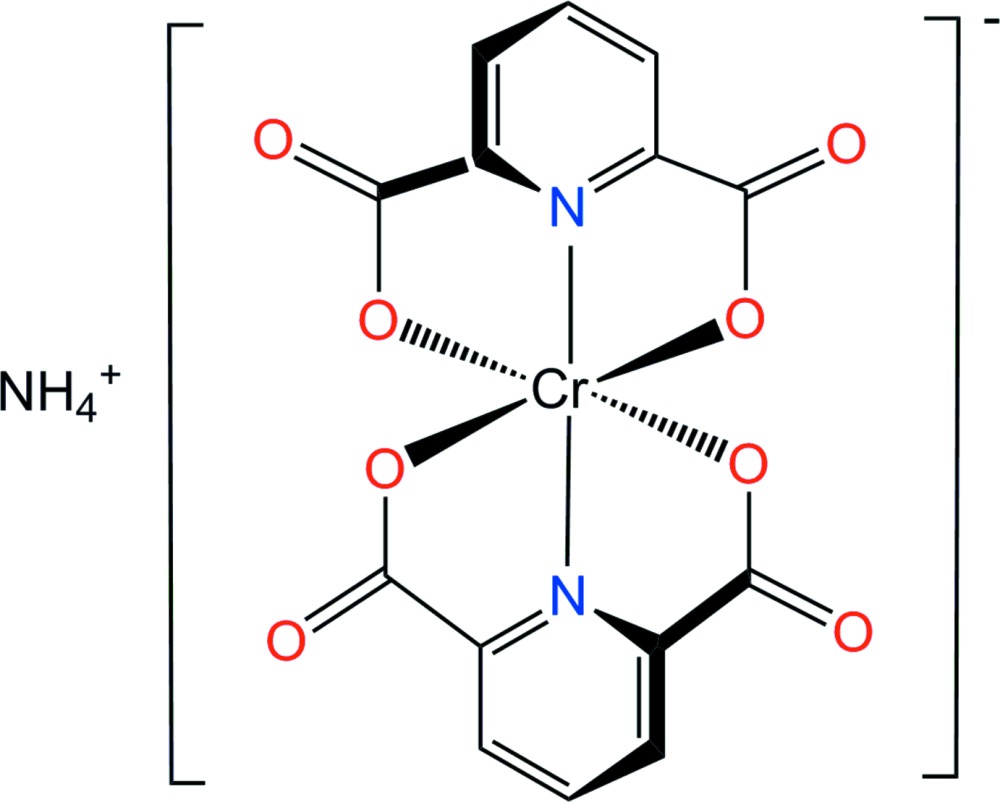


Here we report the crystal structure of (NH_4_)[Cr(pydc)_2_] in order to clarify unambiguously the bonding mode of the two pyridine-2,6-di­carboxyl­ato ligands and the structural arrangement of this ammonium salt.

## Structural commentary   

**Figure 1:**
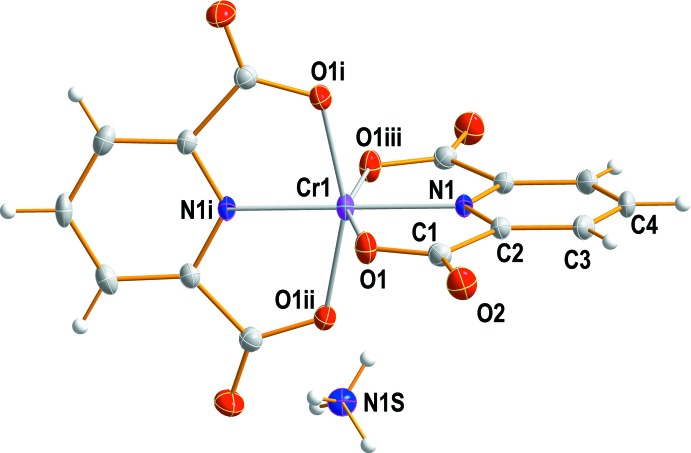
The mol­ecular structure of (NH_4_)[Cr(pydc)_2_], showing the atom-numbering scheme. Non-H atoms are shown as displacement ellipsoids drawn at the 50% probability level.

Counter-ionic species play a very important role in coordin­ation chemistry. The structure reported here is another example of a [Cr(pydc)_2_]^−^ salt but with a different cation. The structural analysis shows that the two tridentate pyridine-2,6-di­carboxyl­ate (pydc) dianions octa­hedrally coordinate to the Cr^III^ metal center through one N atom and two carboxyl­ate O atoms in a meridional arrangement. The Cr^III^ ion is located on a crystallographic fourfold rotoinversion axis (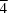). An ellipsoid plot of title complex together with the atomic numbering is illustrated in Fig. 1 ▸.

The Cr—N and Cr—O bond lengths to the pydc^2−^ ligands are 1.9727 (15) and 1.9889 (9) Å, respectively, and these lengths agree well with the values observed in the literature for complexes with the same [Cr(pydc)_2_]^−^ anion (Fürst *et al.*, 1979 ▸; Dai *et al.*, 2006 ▸; González-Baró *et al.*, 2008 ▸; Zhou *et al.*, 2009 ▸; Hakimi *et al.*, 2012 ▸). The coordinating pyridine N atoms are in a mutually *trans* arrangement. Both tridendate pydc^2−^ ligands are nearly planar and are oriented perpendicular to one another. Bond angles about the central chromium atom are 79.10 (3) for N1—Cr1—O1, 100.90 (3) for N1—Cr1—O1^i^ and 158.20 (5)° for O1^i^—Cr1—O1^ii^, indicating a distorted octa­hedral coordination environment [symmetry codes: (i) −*y* + 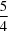, *x* − 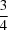, −*z* + 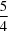; (ii) *y* + 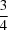, −*x* + 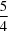, −*z* + 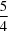]. The C1—O1 and C1—O2 bond lengths within the carboxyl­ate group of the pydc^2−^ ligand are 1.2941 (15) and 1.2223 (14) Å, respectively, and can be compared with values of 1.298 (5) and 1.224 (5) Å for Rb[Cr(pydc)_2_] (Fürst *et al.*, 1979 ▸). The ammonium cation, also lying on a crystallographic fourfold rotoinversion axis (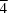), shows a distorted tetra­hedral geometry of the hydrogen atoms around the central nitro­gen atom with N—H distances of 0.846 (9) Å and the H—N—H angles ranging from 105.36 (9) to 118.06 (9)°.

## Supra­molecular features   

**Figure 2:**
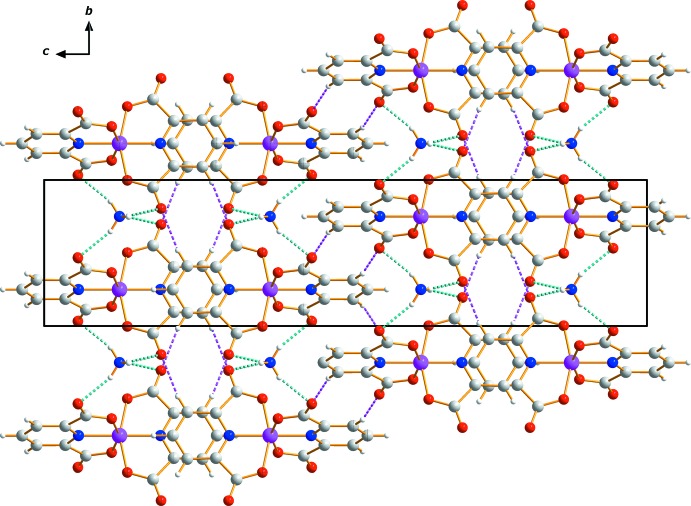
Crystal packing of (NH_4_)[Cr(pydc)_2_], viewed perpendicular to the *bc* plane. Dashed lines represent C—H⋯O (purple) and N—H⋯O (blue) hydrogen-bonding inter­actions.

The pattern of hydrogen bonding around the cation is very similar to the coordination environment in the related potassium salt (Hakimi *et al.*, 2012 ▸). The non-coordinating carbonyl O atom forms weak C—H⋯O hydrogen bonds that contribute to the crystal packing. The ammonium cation is also linked to the carbonyl O atoms from four neighboring pydc^2−^ ligands through classical N—H⋯O hydrogen bonds (Table 1 ▸). An extensive array of these contacts generate a three-dimensional network of mol­ecules stacked along the *a-*axis direction (Fig. 2 ▸). π–π inter­actions involving adjacent pyridine rings further link the components of the structure into a three-dimensional network. The centroid–centroid distance between the π–π stacked rings (N1/C2–C4/C3^iv^/C2^iv^)⋯(N1^v^/C2^v^–C4^v^/C3^vi^/C2^vi^) is 3.572 (2) Å [symmetry codes: (iv) 2 − *x*, 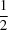 − *y*, *z*; (v) 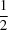 + *x*, *y*, 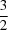 − *z*; (vi) 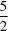 − *x*, 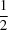 − *y*, 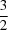 − *z*].

## Database survey   

A search of the Cambridge Structural Database (Version 5.35, May 2014 with one update; Groom & Allen, 2014 ▸) indicates a total of 16 hits for Cr^III^ complexes with a complex anion [Cr(pydc)_2_]^−^ unit. Many crystal structures of [Cr(pydc)_2_]^−^ with inorganic, organic or complex counter-cations such as K^+^ (Hakimi *et al.*, 2012 ▸), Na^+^ (Dai *et al.*, 2006 ▸; González-Baró *et al.*, 2008 ▸; Zhou *et al.*, 2009 ▸), Rb^+^ (Fürst *et al.*, 1979 ▸), creatH^+^ (creat = creatinine; Aghabozorg *et al.*, 2008 ▸), 4,4′-bpyH^+^ (bpy = bi­pyridine; Soleimannejad *et al.*, 2008 ▸), dmpH^+^ (dmp = 2,9-dimethyl-1,10-phenanthrone; Aghajani *et al.*, 2009 ▸), 2-apymH^+^ (2-apym = 2-amino­pyrimidine; Eshtiagh-Hosseini *et al.*, 2010 ▸), [Cr(tpy)(pydc)]^+^ [tpy = 2,6-bis­(2-pyrid­yl)pyridine; Casellato *et al.*, 1991 ▸] and [Ag(atr)_2_]^+^ (atr = 3-amino-1*H*-1,2,4-triazole; Tabatabaee *et al.*, 2011 ▸) have been determined.

Alternative coordination behaviors of the pydc ligands are found in [Cu(Hpydc)_2_]·3H_2_O, which has one neutral H_2_pydc and one divalent pydc^2−^ ligand, while [Ni(Hpydc)_2_]·3H_2_O (Nathan & Mai, 2000 ▸) has two meridional univalent Hpydc^−^ ligands. The ligands in [Ni(cyclam)(Hpydc)_2_]·2H_2_O (Park *et al.*, 2007 ▸) and Na_2_[Pt(Hpydc)_2_]·6H_2_O are monodentate and bidentate, respectively, while Hpydc^−^ is tridentate in the complexes [Cu(Hpydc)_2_]·3H_2_O and [Ni(Hpydc)_2_]·3H_2_O (Nathan & Mai, 2000 ▸).

## Synthesis and crystallization   

All chemicals were reagent-grade materials and were used without further purification. The starting material, Na[Cr(pydc)_2_]·2H_2_O was prepared as described previously (Hoggard & Schmidtke, 1973 ▸). The sodium salt (0.20 g) was dissolved in 15 mL of water at 323 K and added to 3 mL of water containing 0.5 g of NH_4_Cl. The resulting solution was filtered and allowed to stand at room temperature for several days to give brown block-like crystals of the ammonium salt NH_4_[Cr(pydc)_2_] suitable for X-ray structural analysis.

## Refinement   

Crystal data, data collection and structure refinement details are summarized in Table 2 ▸. All H atoms were placed in geometrically idealized positions and constrained to ride on their parent atoms, with C—H distances of 0.95 (ring H atoms) and with *U*_iso_(H) = 1.2 *U*_eq_(parent atom). The H atoms of the ammonium cation were located from difference Fourier maps and refined with restraints and a fixed N—H distance of 0.87 Å, with *U*_iso_(H) = 1.2*U*_eq_(N). One reflection with *F*_o_<<<*F*_c_ was omitted from the final refinement cycles. The slightly low fraction of measured reflections results from the geometry of the 2D-SMC beamline goniostat.

## Supplementary Material

Crystal structure: contains datablock(s) I. DOI: 10.1107/S2056989015001152/sj5438sup1.cif

Structure factors: contains datablock(s) I. DOI: 10.1107/S2056989015001152/sj5438Isup2.hkl

CCDC reference: 1044324

Additional supporting information:  crystallographic information; 3D view; checkCIF report

## Figures and Tables

**Table 1:** Hydrogen-bond geometry (, )

*D*H*A*	*D*H	H*A*	*D* *A*	*D*H*A*
C3H3O2^i^	0.93	2.50	3.4071(15)	167
N1*S*H1*S*O2^ii^	0.85(1)	2.04(1)	2.8462(11)	158(2)

Symmetry codes: (i) 
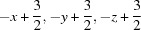
; (ii) 
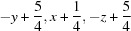
.

**Table 2:** Experimental details

Crystal data	
Chemical formula	(NH_4_)[Cr(C_7_H_3_NO_4_)_2_]
*M*_r_	400.25
Crystal system, space group	Tetragonal, *I*4_1_/*a*
Temperature (K)	301
*a*, *c* ()	7.0305(10), 28.995(6)
*V* (^3^)	1433.2(5)
*Z*	4
Radiation type	Synchrotron, = 0.62998
(mm^1^)	0.62
Crystal size (mm)	0.15 0.10 0.10

Data collection	
Diffractometer	ADSC Q210 CCD area detector
Absorption correction	Empirical (using intensity measurements) (*HKL3000sm *SCALEPACK**; Otwinowski Minor, 1997 ▸)
*T*_min_, *T*_max_	0.925, 0.940
No. of measured, independent and observed [*I* > 2(*I*)] reflections	6841, 943, 903
*R*_int_	0.052
(sin /)_max_ (^1^)	0.695

Refinement	
*R*[*F* ^2^ > 2(*F* ^2^)], *wR*(*F* ^2^), *S*	0.028, 0.081, 1.21
No. of reflections	943
No. of parameters	63
No. of restraints	1
H-atom treatment	H atoms treated by a mixture of independent and constrained refinement
_max_, _min_ (e ^3^)	0.31, 0.86

Computer programs: *PAL ADSC Quantum-210 ADX* (Arvai Nielsen, 1983 ▸), *HKL3000sm* (Otwinowski Minor, 1997 ▸), *SHELXT2014/5* (Sheldrick, 2015*a* ▸), *SHELXL2014/7* (Sheldrick, 2008 ▸, 2015*b* ▸), *DIAMOND* (Brandenburg, 2007 ▸) and *publCIF* (Westrip 2010 ▸).

## supplementary crystallographic information

### Crystal data

**Table:**

(NH_4_)[Cr(C_7_H_3_NO_4_)_2_]	*D*_x_ = 1.855 Mg m^−^^3^
*M**_r_* = 400.25	Synchrotron radiation, λ = 0.62998 Å
Tetragonal, *I*4_1_/*a*	Cell parameters from 20017 reflections
*a* = 7.0305 (10) Å	θ = 0.4–33.6°
*c* = 28.995 (6) Å	µ = 0.62 mm^−^^1^
*V* = 1433.2 (5) Å^3^	*T* = 301 K
*Z* = 4	Block, brown
*F*(000) = 812	0.15 × 0.10 × 0.10 mm

### Data collection

**Table:**

ADSC Q210 CCD area detector diffractometer	903 reflections with *I* > 2σ(*I*)
Radiation source: PLSII 2D bending magnet	*R*_int_ = 0.052
ω scan	θ_max_ = 26.0°, θ_min_ = 4.0°
Absorption correction: empirical (using intensity measurements) (*HKL3000sm *SCALEPACK**; Otwinowski & Minor, 1997)	*h* = −9→9
*T*_min_ = 0.925, *T*_max_ = 0.940	*k* = −9→9
6841 measured reflections	*l* = −36→36
943 independent reflections	

### Refinement

**Table:**

Refinement on *F*^2^	1 restraint
Least-squares matrix: full	Hydrogen site location: mixed
*R*[*F*^2^ > 2σ(*F*^2^)] = 0.028	H atoms treated by a mixture of independent and constrained refinement
*wR*(*F*^2^) = 0.081	*w* = 1/[σ^2^(*F*_o_^2^) + (0.0461*P*)^2^ + 0.7347*P*] where *P* = (*F*_o_^2^ + 2*F*_c_^2^)/3
*S* = 1.21	(Δ/σ)_max_ = 0.001
943 reflections	Δρ_max_ = 0.31 e Å^−^^3^
63 parameters	Δρ_min_ = −0.86 e Å^−^^3^

### Special details

**Table:**

Geometry. All esds (except the esd in the dihedral angle between two l.s. planes) are estimated using the full covariance matrix. The cell esds are taken into account individually in the estimation of esds in distances, angles and torsion angles; correlations between esds in cell parameters are only used when they are defined by crystal symmetry. An approximate (isotropic) treatment of cell esds is used for estimating esds involving l.s. planes.

### Fractional atomic coordinates and isotropic or equivalent isotropic displacement parameters (Å^2^)

**Table:**

	*x*	*y*	*z*	*U*_iso_*/*U*_eq_	
Cr1	1.0000	0.2500	0.6250	0.01282 (14)	
O1	0.88412 (13)	0.50247 (12)	0.63797 (3)	0.0205 (2)	
O2	0.78279 (14)	0.69538 (13)	0.69398 (4)	0.0261 (2)	
N1	1.0000	0.2500	0.69303 (5)	0.0133 (3)	
C1	0.86229 (15)	0.55077 (15)	0.68071 (4)	0.0163 (2)	
C2	0.93527 (14)	0.40403 (15)	0.71495 (4)	0.0142 (2)	
C3	0.93425 (15)	0.41005 (17)	0.76260 (4)	0.0189 (2)	
H3	0.8912	0.5171	0.7783	0.023*	
C4	1.0000	0.2500	0.78645 (6)	0.0201 (3)	
H4	1.0000	0.2500	0.8185	0.024*	
N1S	0.5000	0.7500	0.6250	0.0223 (4)	
H1S	0.502 (3)	0.8529 (19)	0.6099 (7)	0.027*	

### Atomic displacement parameters (Å^2^)

**Table:**

	*U*^11^	*U*^22^	*U*^33^	*U*^12^	*U*^13^	*U*^23^
Cr1	0.01591 (16)	0.01591 (16)	0.0066 (2)	0.000	0.000	0.000
O1	0.0286 (4)	0.0193 (4)	0.0134 (5)	0.0055 (3)	−0.0013 (3)	0.0013 (3)
O2	0.0304 (5)	0.0222 (4)	0.0258 (5)	0.0098 (3)	−0.0009 (3)	−0.0037 (3)
N1	0.0146 (5)	0.0171 (6)	0.0082 (7)	0.0008 (4)	0.000	0.000
C1	0.0159 (4)	0.0170 (5)	0.0160 (6)	0.0004 (3)	−0.0013 (4)	−0.0005 (4)
C2	0.0133 (4)	0.0175 (4)	0.0117 (5)	0.0001 (3)	−0.0005 (3)	−0.0016 (3)
C3	0.0171 (5)	0.0258 (5)	0.0137 (6)	−0.0006 (4)	0.0005 (3)	−0.0059 (4)
C4	0.0199 (7)	0.0324 (8)	0.0078 (8)	−0.0021 (5)	0.000	0.000
N1S	0.0220 (6)	0.0220 (6)	0.0229 (12)	0.000	0.000	0.000

### Geometric parameters (Å, º)

**Table:**

Cr1—N1	1.9727 (15)	N1—C2	1.3355 (12)
Cr1—N1^i^	1.9727 (15)	C1—C2	1.5208 (15)
Cr1—O1	1.9889 (9)	C2—C3	1.3824 (16)
Cr1—O1^ii^	1.9889 (9)	C3—C4	1.3993 (15)
Cr1—O1^i^	1.9889 (9)	C3—H3	0.9300
Cr1—O1^iii^	1.9889 (9)	C4—C3^iii^	1.3992 (15)
O1—C1	1.2941 (15)	C4—H4	0.9300
O2—C1	1.2223 (14)	N1S—H1S	0.846 (9)
N1—C2^iii^	1.3354 (12)		
			
N1—Cr1—N1^i^	180.0	C2^iii^—N1—C2	123.19 (15)
N1—Cr1—O1	79.10 (3)	C2^iii^—N1—Cr1	118.40 (7)
N1^i^—Cr1—O1	100.90 (3)	C2—N1—Cr1	118.41 (7)
N1—Cr1—O1^ii^	100.90 (3)	O2—C1—O1	125.02 (11)
N1^i^—Cr1—O1^ii^	79.10 (3)	O2—C1—C2	120.87 (11)
O1—Cr1—O1^ii^	92.049 (10)	O1—C1—C2	114.02 (9)
N1—Cr1—O1^i^	100.90 (3)	N1—C2—C3	120.14 (11)
N1^i^—Cr1—O1^i^	79.10 (3)	N1—C2—C1	110.77 (10)
O1—Cr1—O1^i^	92.049 (10)	C3—C2—C1	129.04 (10)
O1^ii^—Cr1—O1^i^	158.20 (5)	C2—C3—C4	117.88 (11)
N1—Cr1—O1^iii^	79.10 (3)	C2—C3—H3	121.1
N1^i^—Cr1—O1^iii^	100.90 (3)	C4—C3—H3	121.1
O1—Cr1—O1^iii^	158.20 (5)	C3^iii^—C4—C3	120.77 (16)
O1^ii^—Cr1—O1^iii^	92.049 (10)	C3^iii^—C4—H4	119.6
O1^i^—Cr1—O1^iii^	92.049 (10)	C3—C4—H4	119.6
C1—O1—Cr1	117.64 (7)		
			
Cr1—O1—C1—O2	−175.18 (9)	O1—C1—C2—N1	−2.76 (12)
Cr1—O1—C1—C2	1.39 (12)	O2—C1—C2—C3	−3.27 (17)
C2^iii^—N1—C2—C3	0.47 (7)	O1—C1—C2—C3	−179.99 (10)
Cr1—N1—C2—C3	−179.53 (7)	N1—C2—C3—C4	−0.90 (14)
C2^iii^—N1—C2—C1	−177.04 (9)	C1—C2—C3—C4	176.10 (9)
Cr1—N1—C2—C1	2.96 (9)	C2—C3—C4—C3^iii^	0.44 (7)
O2—C1—C2—N1	173.96 (9)		

Symmetry codes: (i) *y*+3/4, −*x*+5/4, −*z*+5/4; (ii) −*y*+5/4, *x*−3/4, −*z*+5/4; (iii) −*x*+2, −*y*+1/2, *z*.

### Hydrogen-bond geometry (Å, º)

**Table:**

*D*—H···*A*	*D*—H	H···*A*	*D*···*A*	*D*—H···*A*
C3—H3···O2^iv^	0.93	2.50	3.4071 (15)	167
N1*S*—H1*S*···O2^v^	0.85 (1)	2.04 (1)	2.8462 (11)	158 (2)

Symmetry codes: (iv) −*x*+3/2, −*y*+3/2, −*z*+3/2; (v) −*y*+5/4, *x*+1/4, −*z*+5/4.
